# Supplementary material for: Timbral effects on consonance disentangle psychoacoustic mechanisms and suggest perceptual origins for musical scales
Source: Nat Commun. 2024 Feb 19;15:1482. doi: 10.1038/s41467-024-45812-z (PMC11258268; doi:10.1038/s41467-024-45812-z)
Supplement: Supplementary file 1 — Supplementary Information [file 41467_2024_45812_MOESM1_ESM.pdf]

Supplementary Information

Timbral effects on consonance disentangle psychoacoustic mechanisms and suggest perceptual origins for musical scales

Raja Marjieh, Peter Harrison, Harin Lee, Fotini Deligiannaki, Nori Jacoby

Supplementary Notes

We provide the reader with an interactive web app to facilitate exploration of the results of our experiments as well as the various models discussed in the present work. The interactive web app can be used to listen to the stimuli from the different studies and to export the data. The web app can be accessed via the following link:

- `https://pmcharrison.gitlab.io/timbre-and-consonance-paper/supplementary.html`

For full reproducibility, we also provide the content of the web app as Supplementary Figures and Movies throughout the paper and the present Supplementary Information, in addition to the exported data and code directories provided as .zip files at the following OSF repository:

- `https://osf.io/83w2b/`

Supplementary Figures

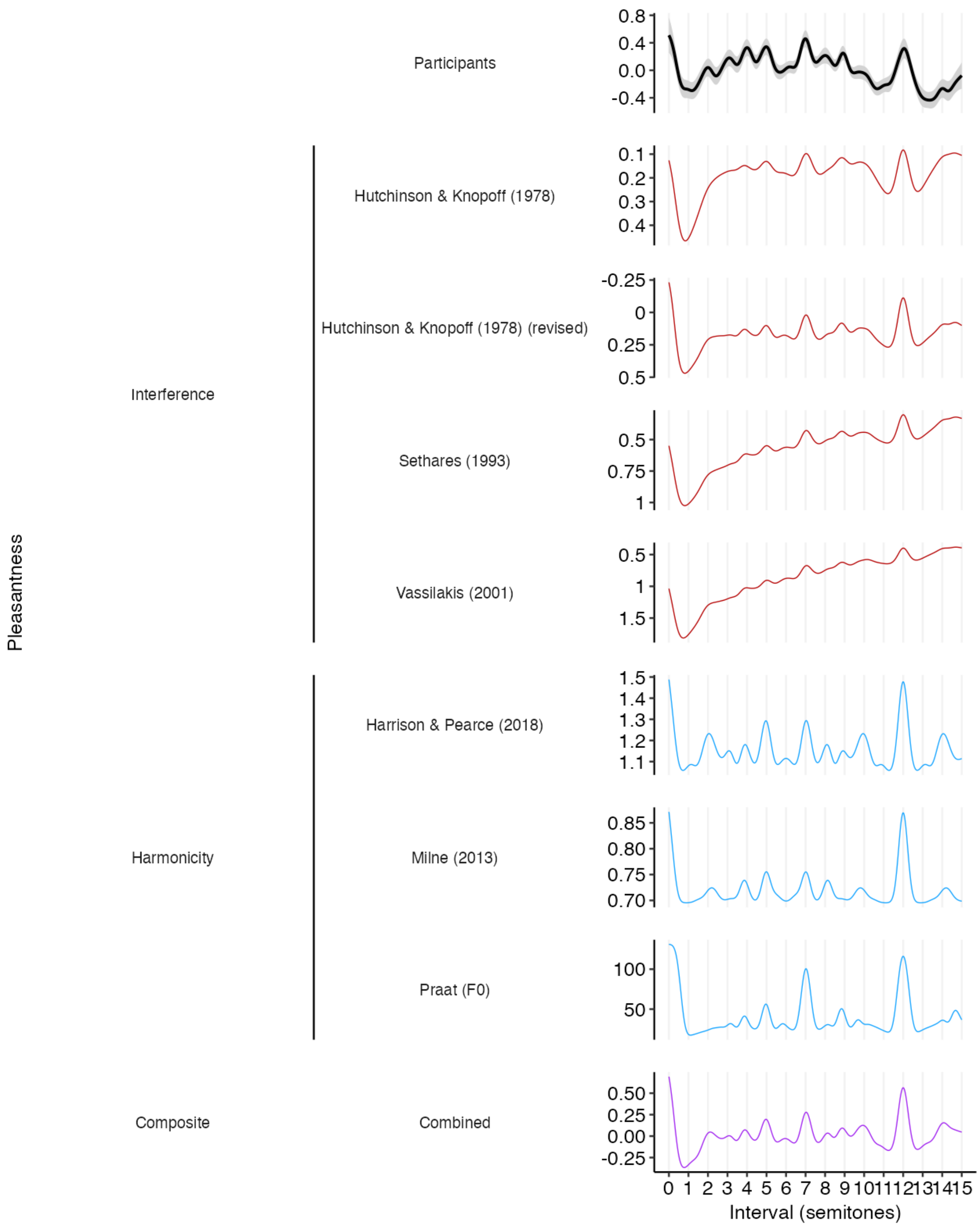

Supplementary Figure 1. Dyadic pleasantness judgments for harmonic complex tones (N = 198 US participants) along with different model predictions. Behavioral results are summarized using a kernel smoother with a bandwidth of 0.2 semitones, with 95% confidence intervals (bootstrapped, 1,000 replicates) shaded in gray.

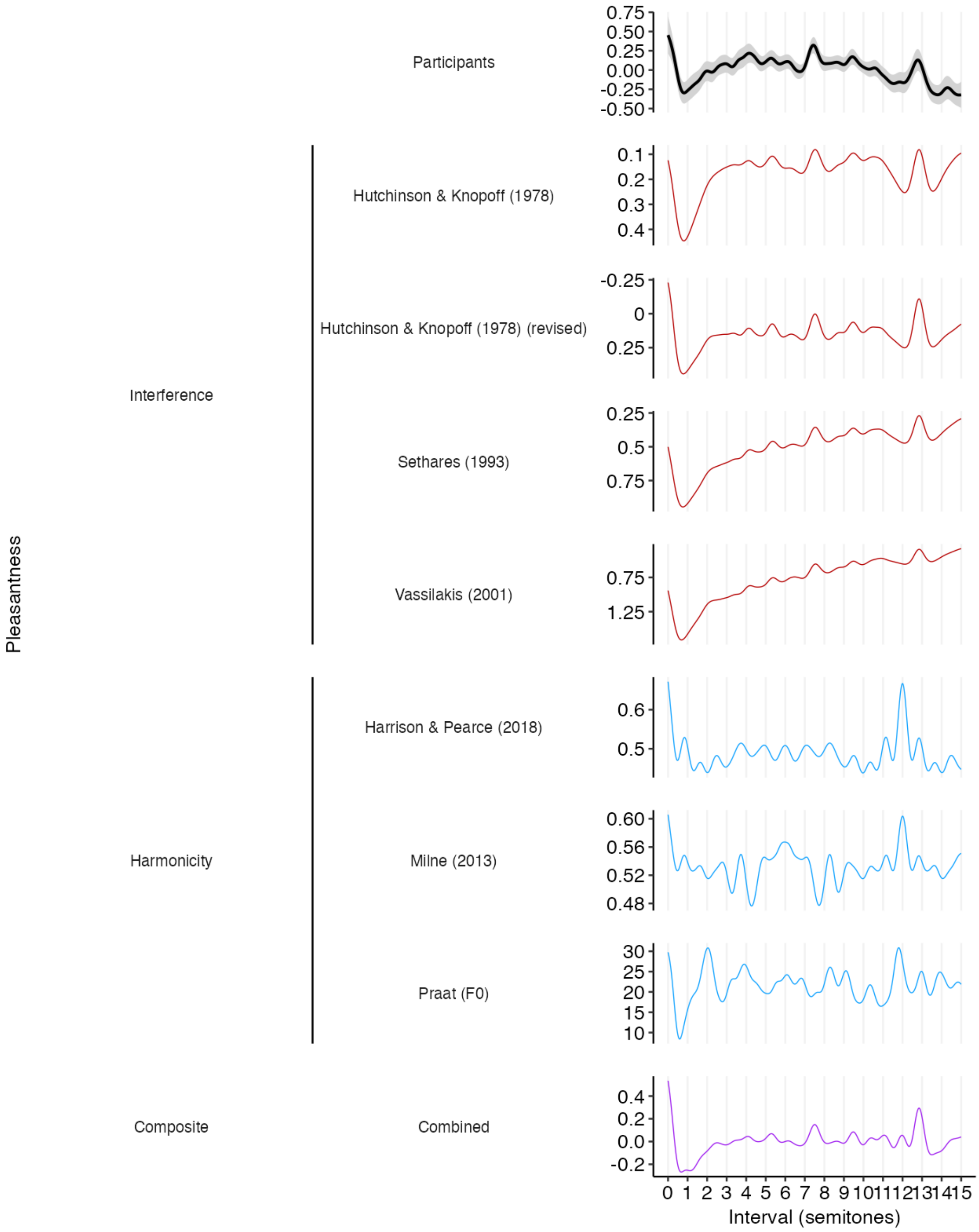

Supplementary Figure 2. Dyadic pleasantness judgments for stretched complex tones (N = 194 US participants) along with different model predictions. Behavioral results are summarized using a kernel smoother with a bandwidth of 0.2 semitones, with 95% confidence intervals (bootstrapped, 1,000 replicates) shaded in gray.

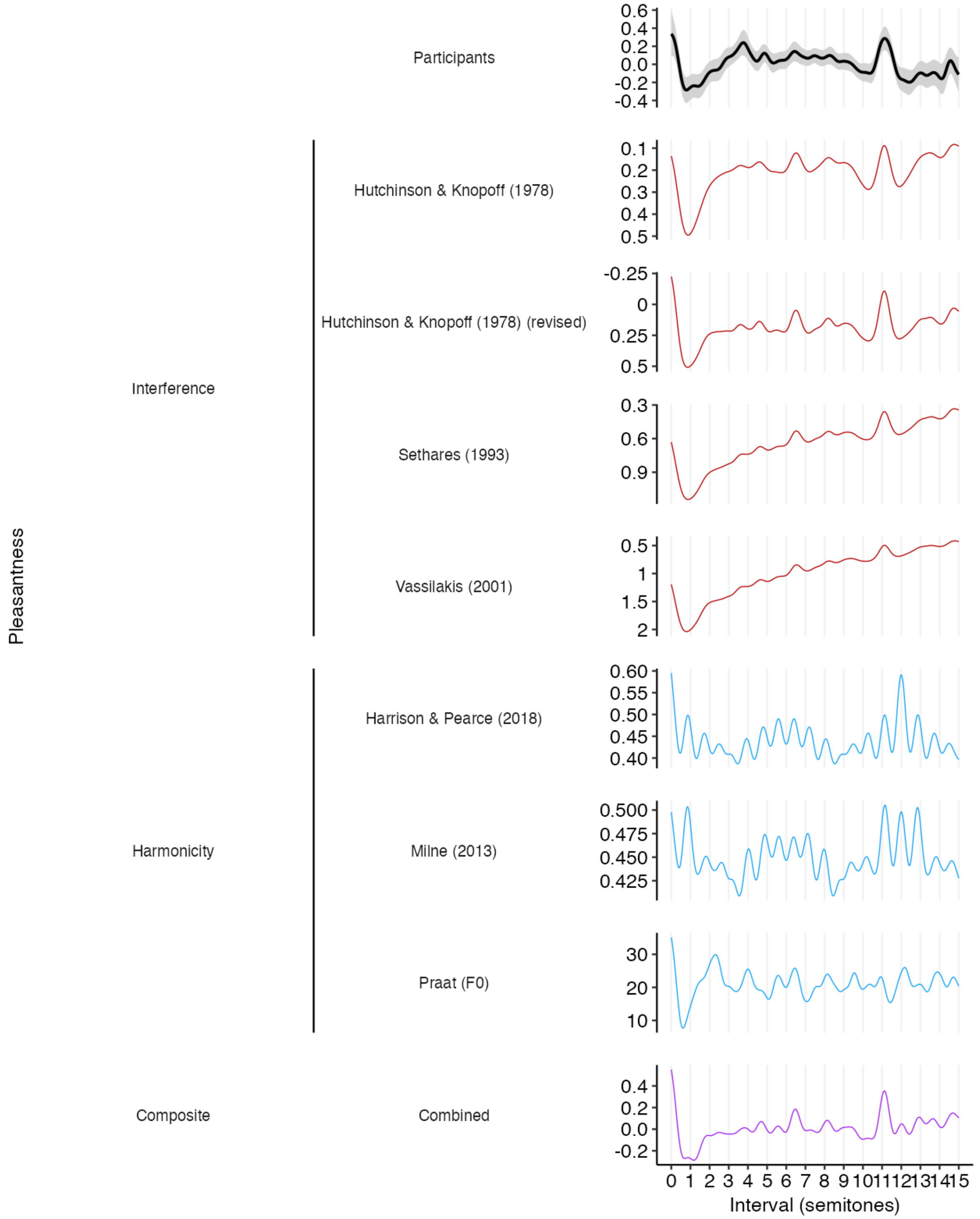

Supplementary Figure 3. Dyadic pleasantness judgments for compressed complex tones (N = 202 US participants) along with different model predictions. Behavioral results are summarized using a kernel smoother with a bandwidth of 0.2 semitones, with 95% confidence intervals (bootstrapped, 1,000 replicates) shaded in gray.

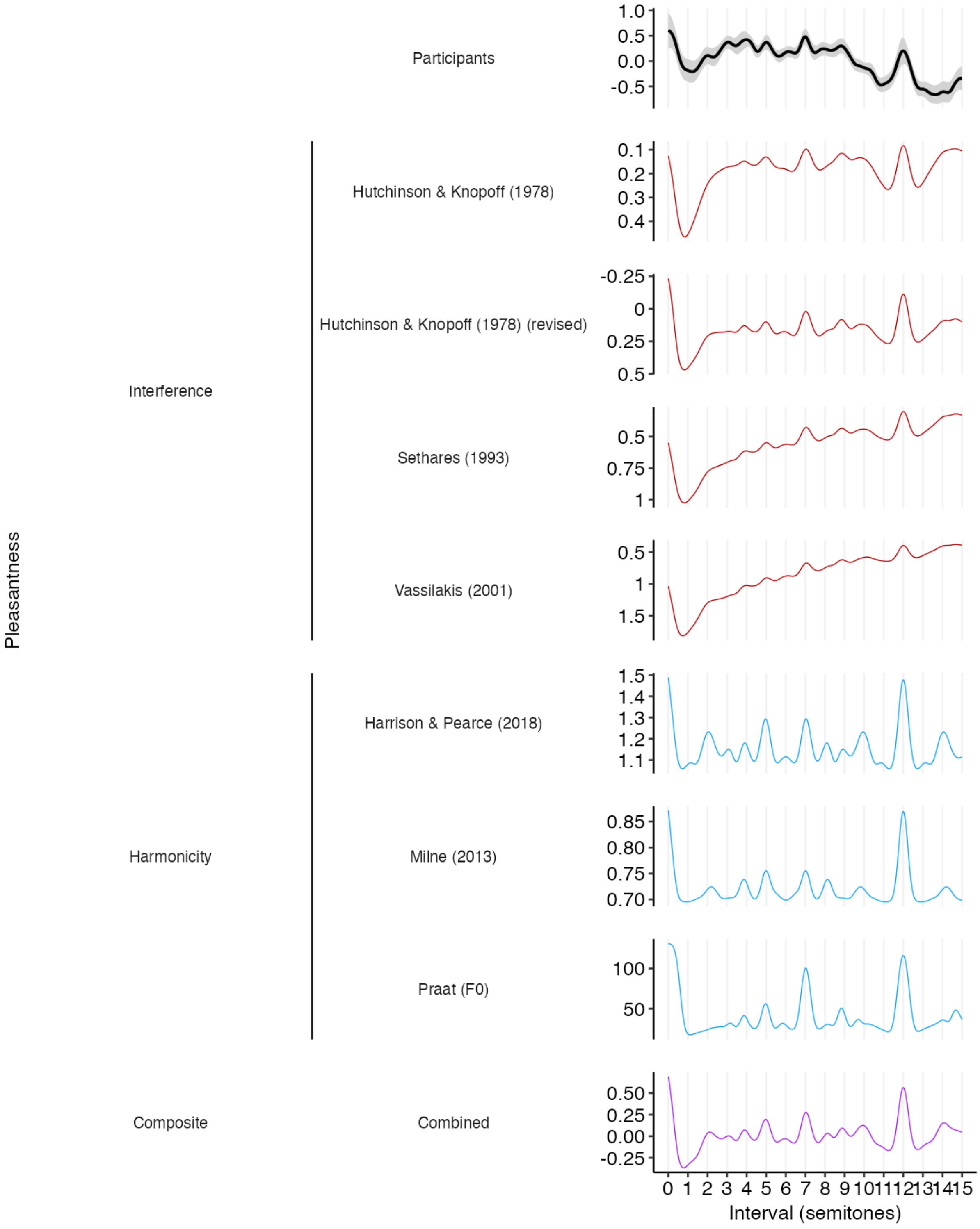

Supplementary Figure 4. Dyadic pleasantness judgments for harmonic complex tones (N = 24 South Korean participants) along with different model predictions. Behavioral results are summarized using a kernel smoother with a bandwidth of 0.2 semitones, with 95% confidence intervals (bootstrapped, 1,000 replicates) shaded in gray.

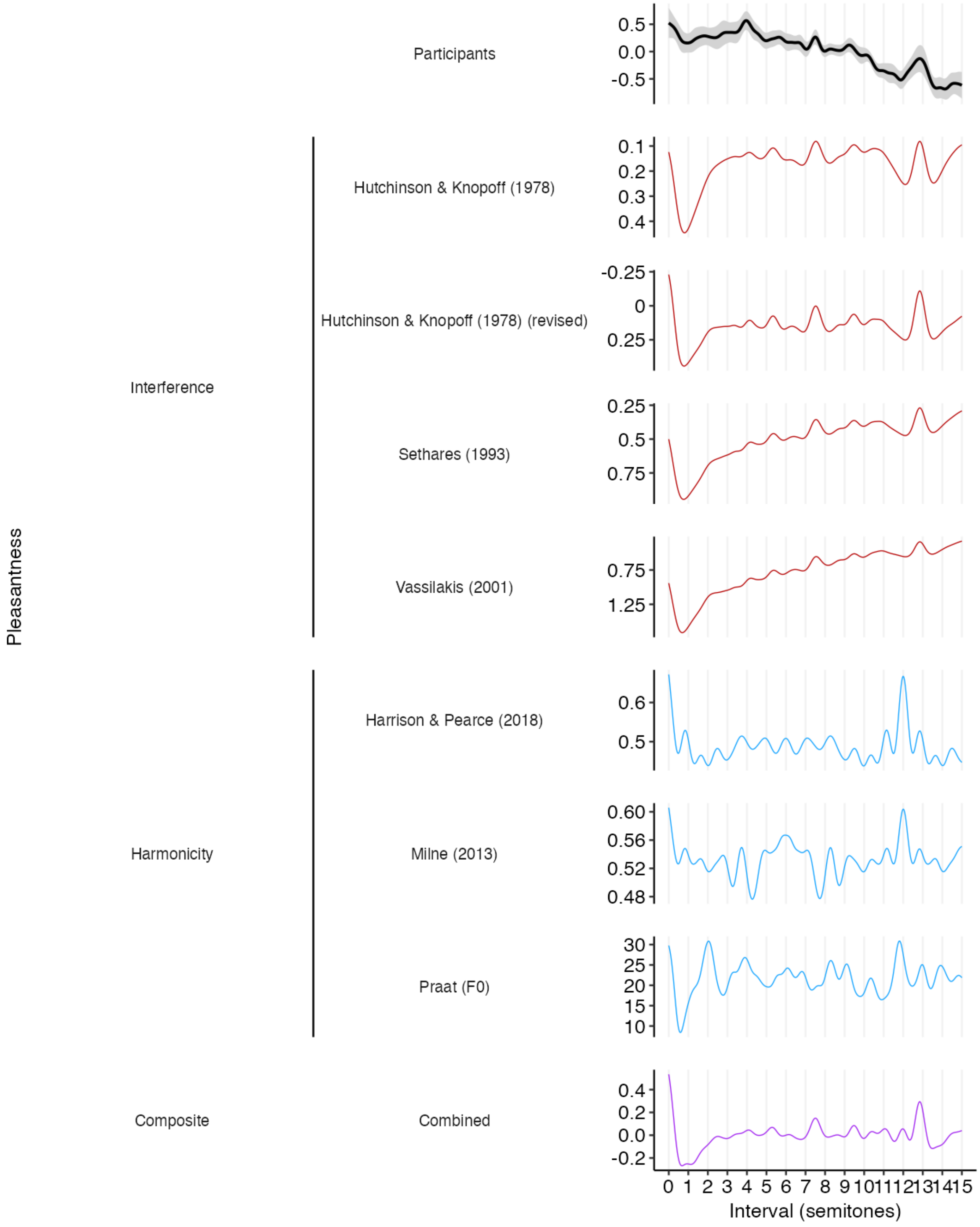

Supplementary Figure 5. Dyadic pleasantness judgments for stretched complex tones (N = 20 South Korean participants) along with different model predictions. Behavioral results are summarized using a kernel smoother with a bandwidth of 0.2 semitones, with 95% confidence intervals (bootstrapped, 1,000 replicates) shaded in gray.

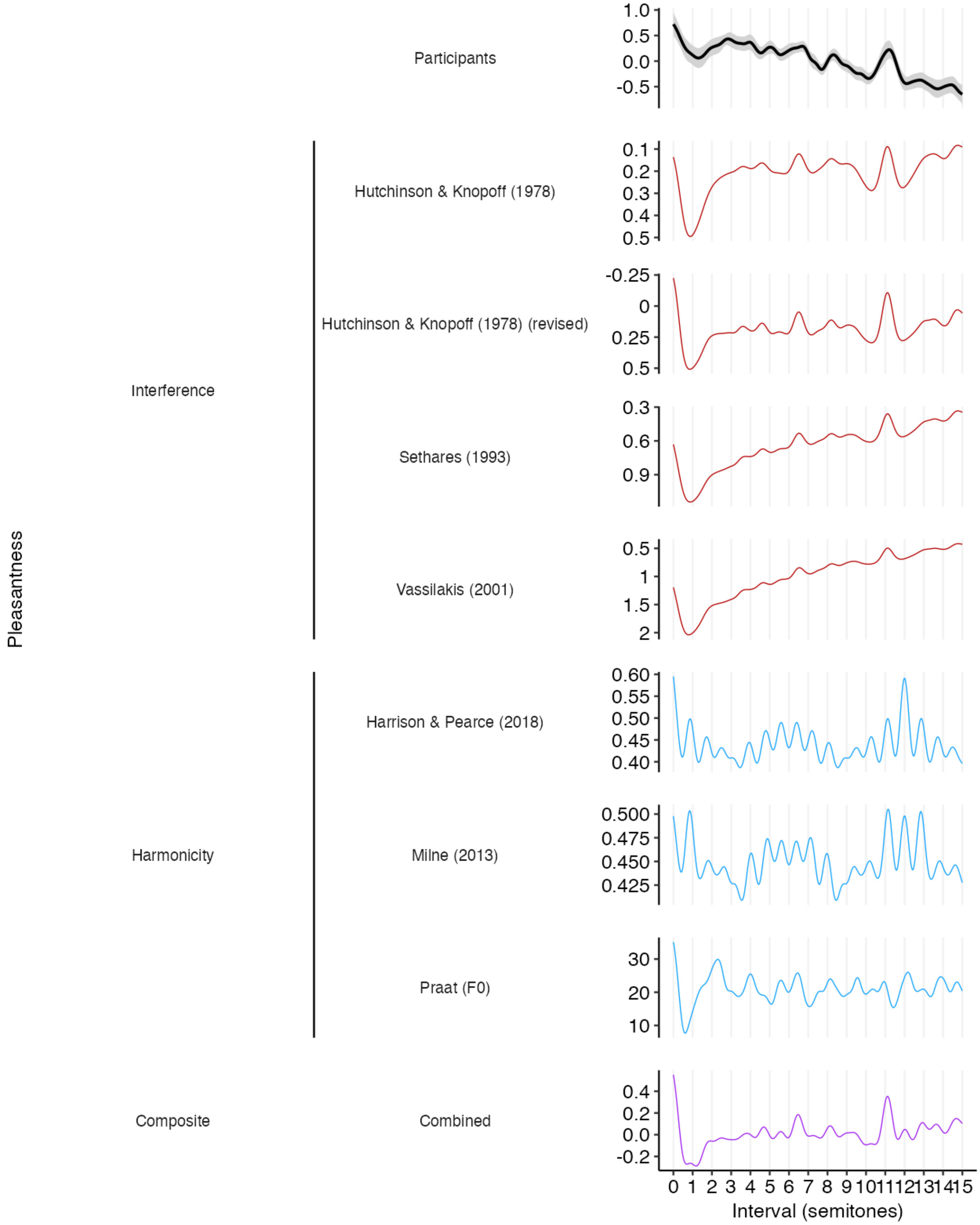

Supplementary Figure 6. Dyadic pleasantness judgments for compressed complex tones (N = 24 South Korean participants) along with different model predictions. Behavioral results are summarized using a kernel smoother with a bandwidth of 0.2 semitones, with 95% confidence intervals (bootstrapped, 1,000 replicates) shaded in gray.

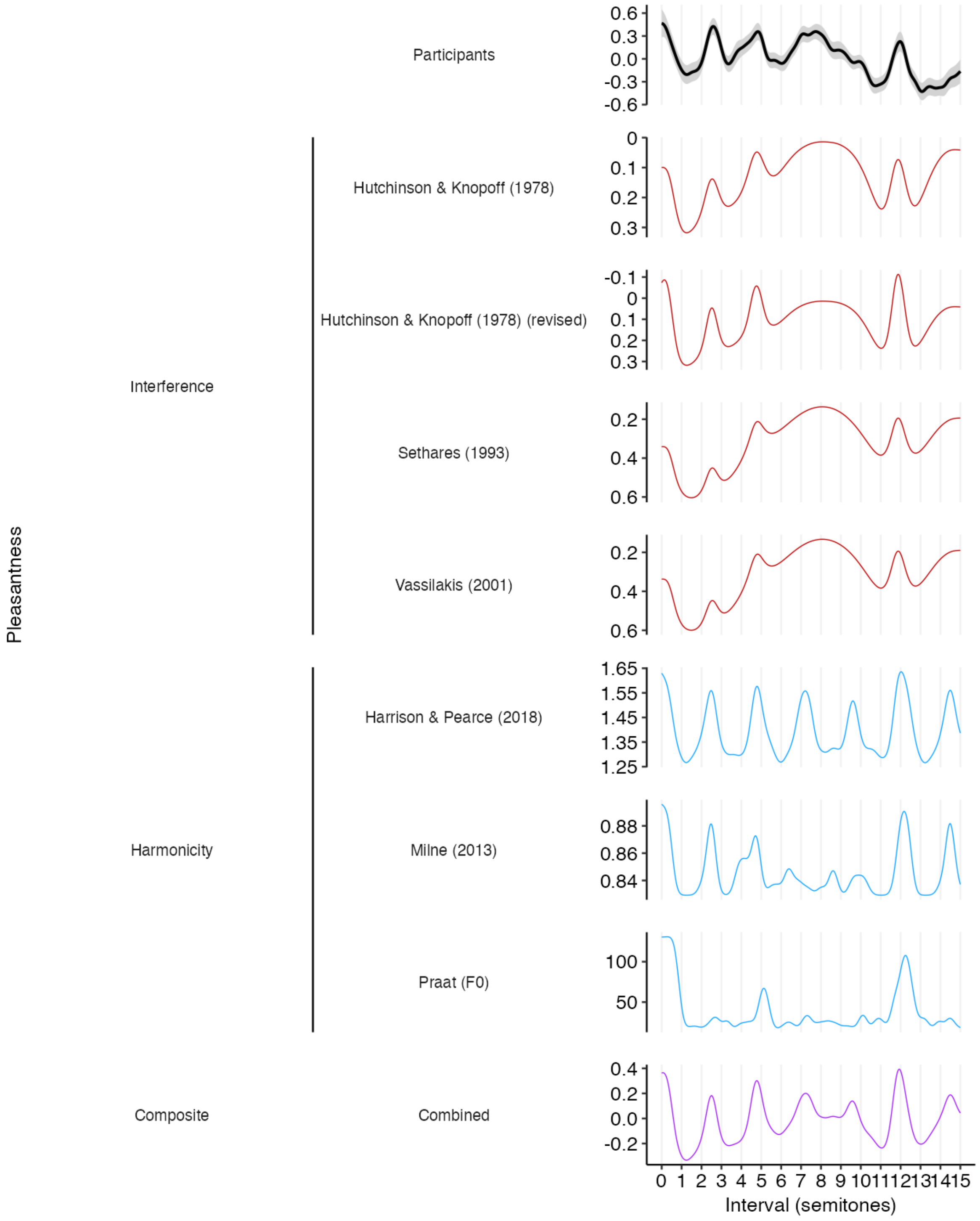

Supplementary Figure 7. Pleasantness judgments for dyads comprising a harmonic complex tone (lower) combined with an idealized bonang tone (upper) (N = 170 US participants) along with model predictions. Behavioral results are summarized using a kernel smoother with a bandwidth of 0.2 semitones, with 95% confidence intervals (bootstrapped, 1,000 replicates) shaded in gray.

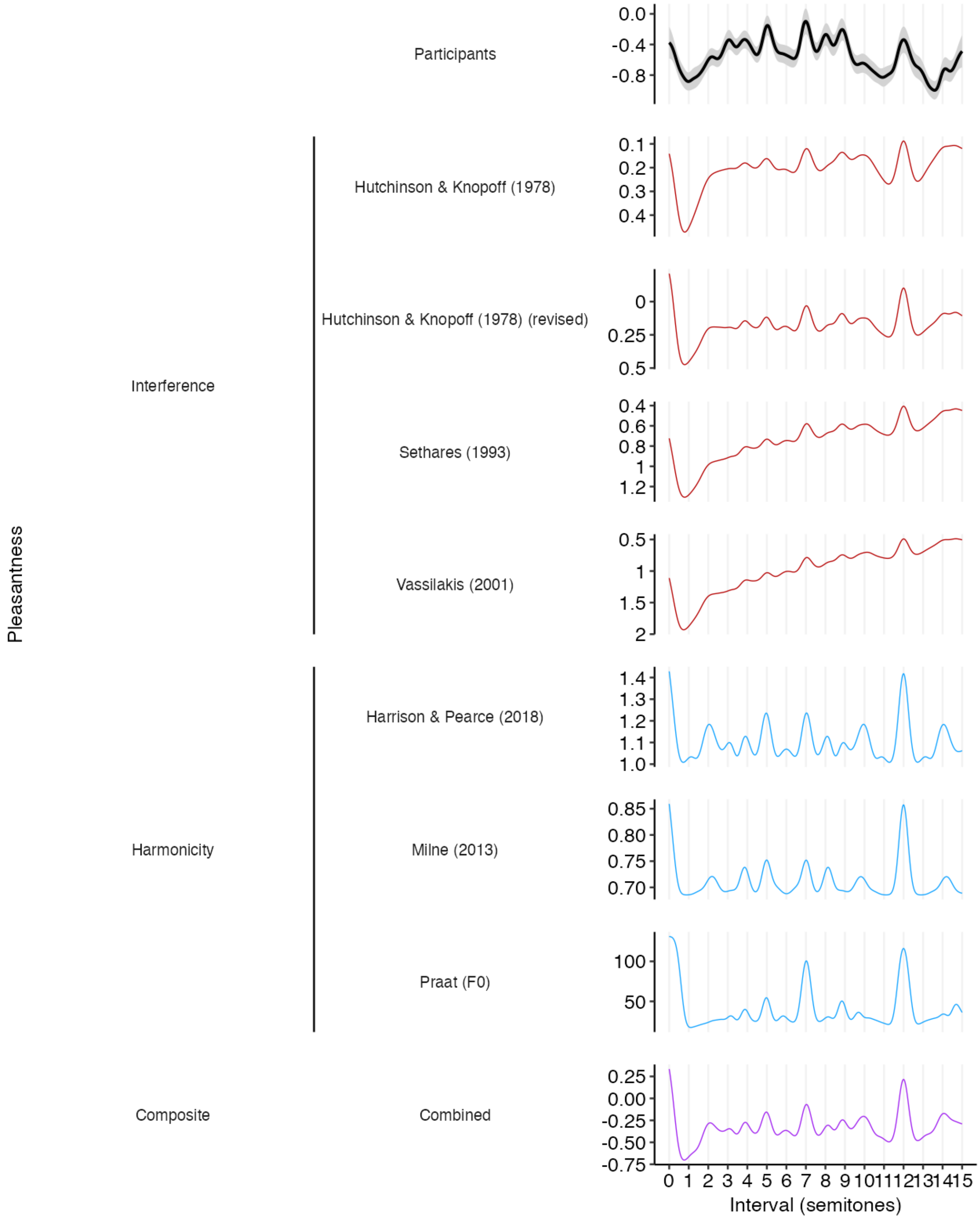

Supplementary Figure 8. Dyadic pleasantness judgments as a function of roll-off (N = 322 US participants) for harmonic dyads with 2 dB/octave roll-off values along with model predictions. Behavioral results are summarized using a kernel smoother with a bandwidth of 0.2 semitones, with 95% confidence intervals (bootstrapped, 1,000 replicates) shaded in gray.

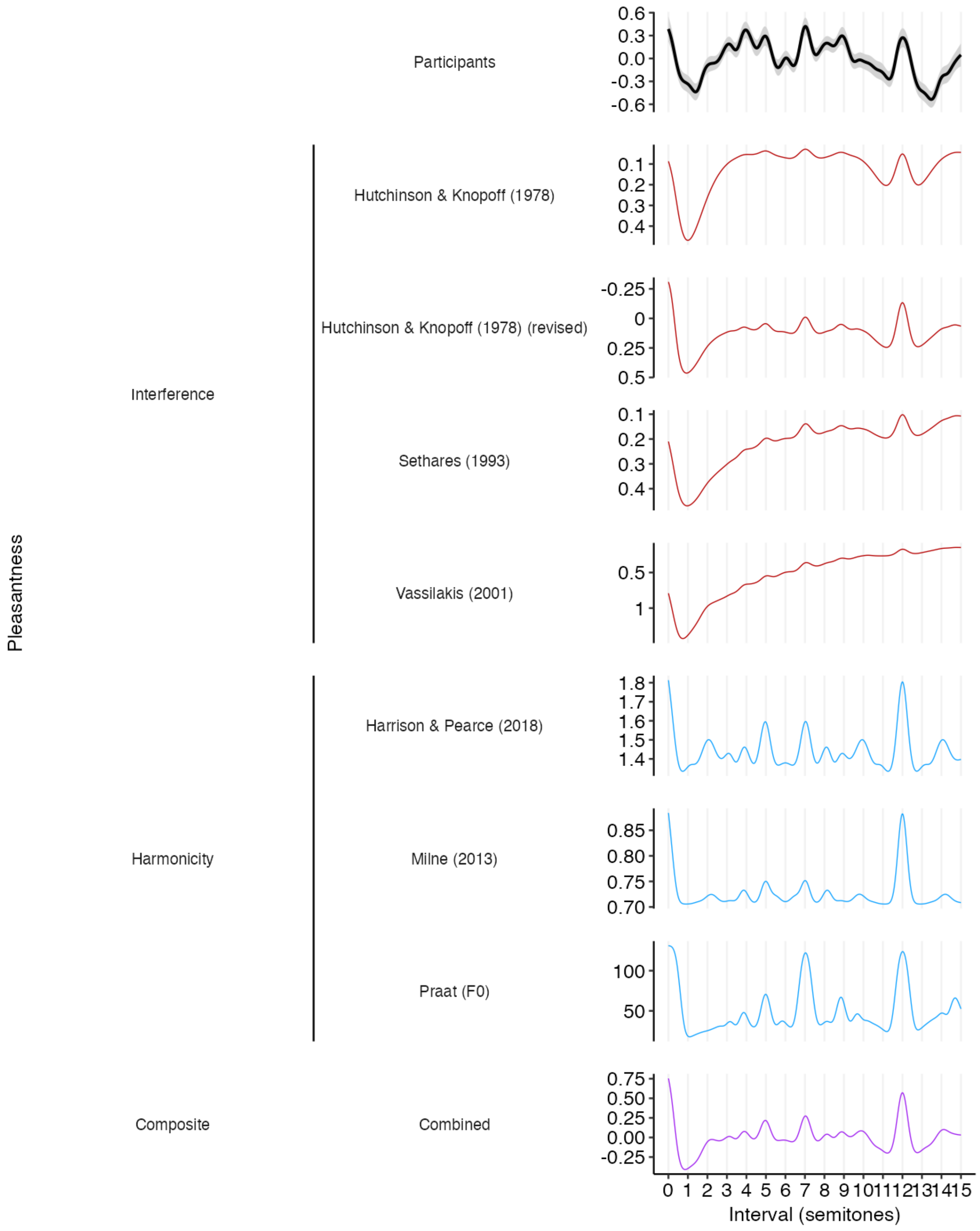

Supplementary Figure 9. Dyadic pleasantness judgments as a function of roll-off (N = 322 US participants) for harmonic dyads with 7 dB/octave roll-off values along with model predictions. Behavioral results are summarized using a kernel smoother with a bandwidth of 0.2 semitones, with 95% confidence intervals (bootstrapped, 1,000 replicates) shaded in gray.

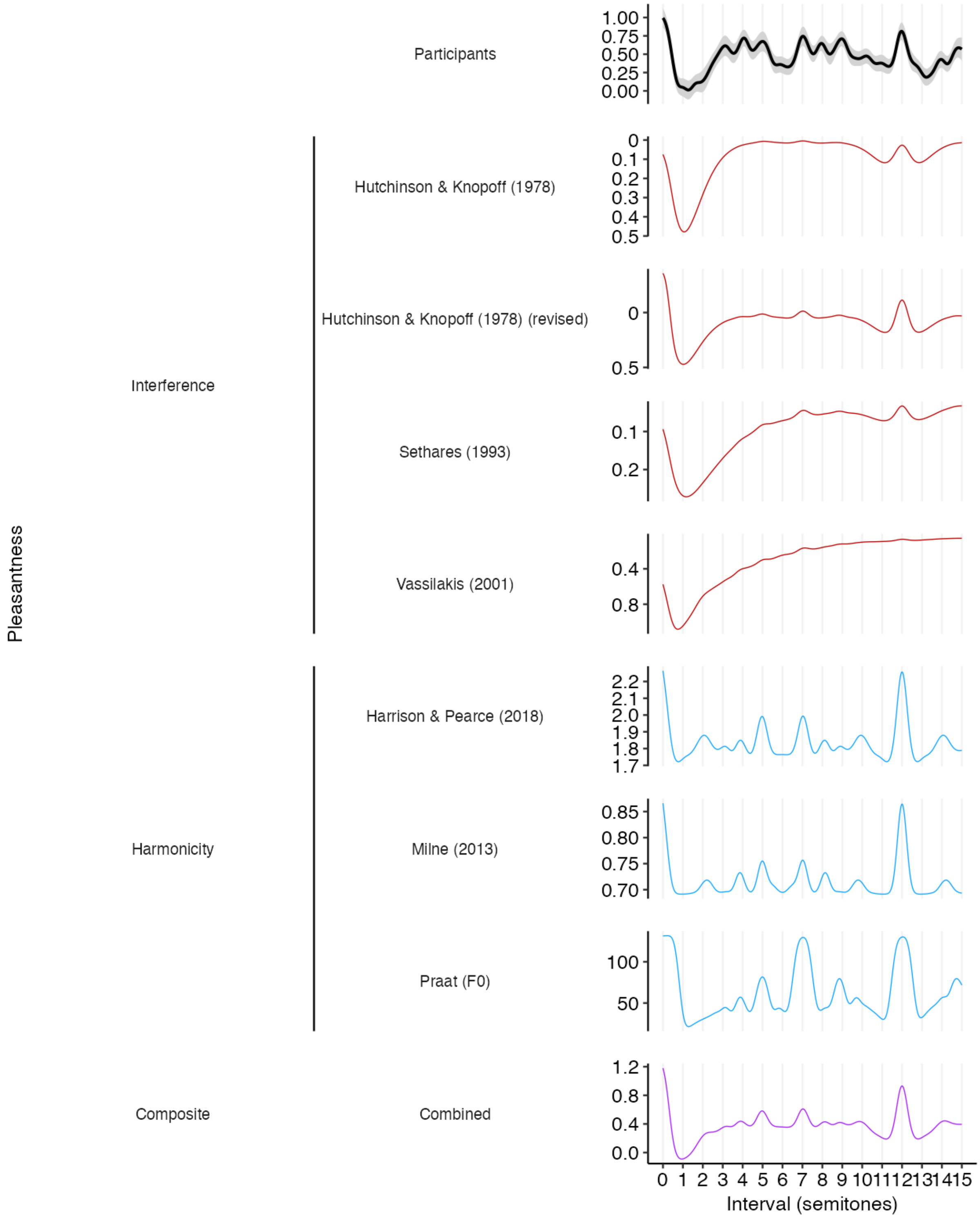

Supplementary Figure 10. Dyadic pleasantness judgments as a function of roll-off (N = 322 US participants) for harmonic dyads with 12 dB/octave roll-off values along with model predictions. Behavioral results are summarized using a kernel smoother with a bandwidth of 0.2 semitones, with 95% confidence intervals (bootstrapped, 1,000 replicates) shaded in gray.

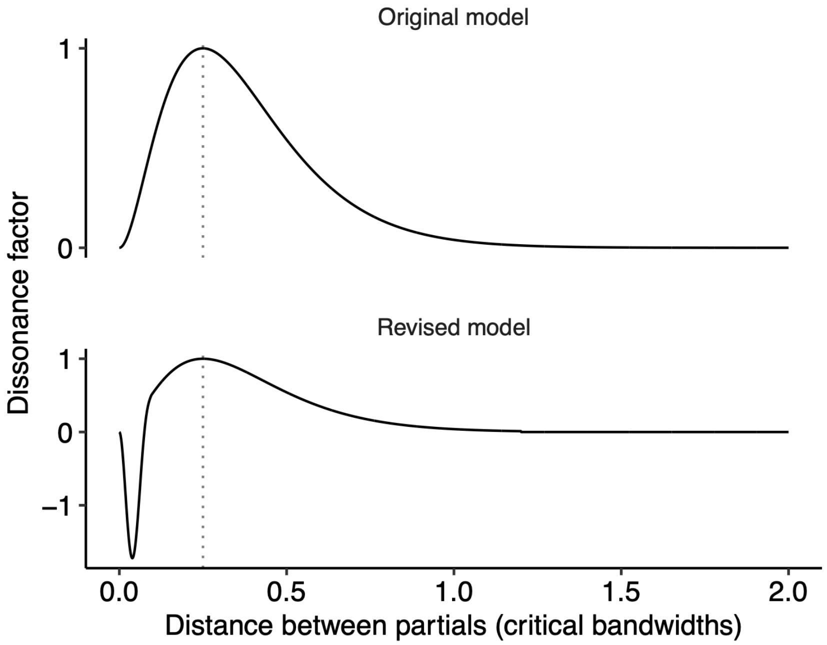

Supplementary Figure 11. The dissonance curve of the Hutchinson-Knopoff model, in both original and revised forms. The distance between partials that achieves maximal dissonance (0.25) is annotated with a dotted line.

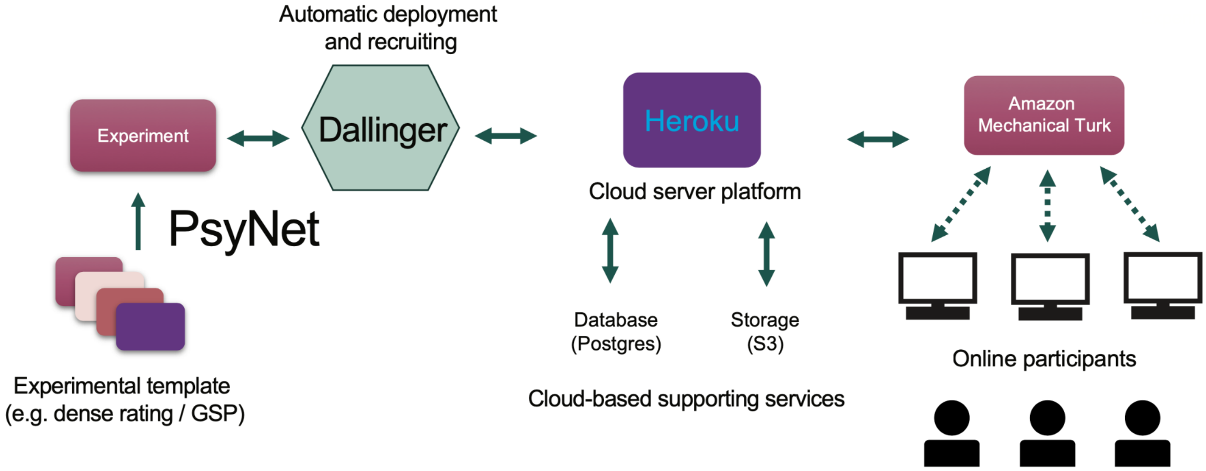

Supplementary Figure 12. Schematic of the data collection infrastructure.

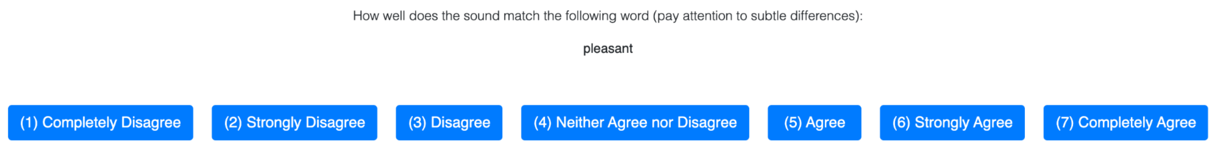

Supplementary Figure 13. Participant interface for a dense rating trial.

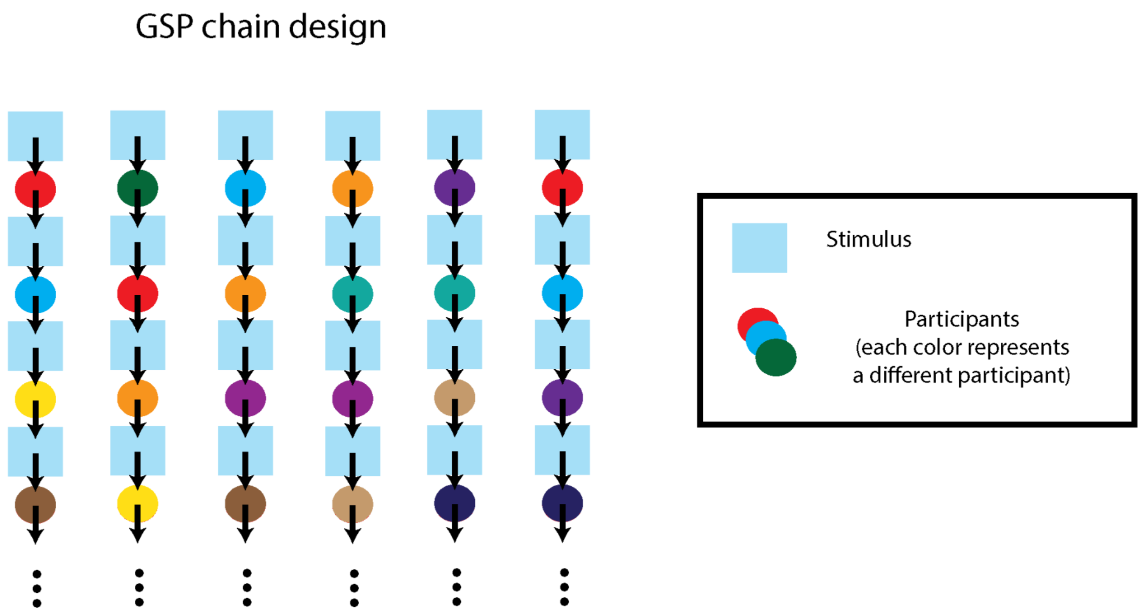

Supplementary Figure 14. Gibbs Sampling with People chain design.

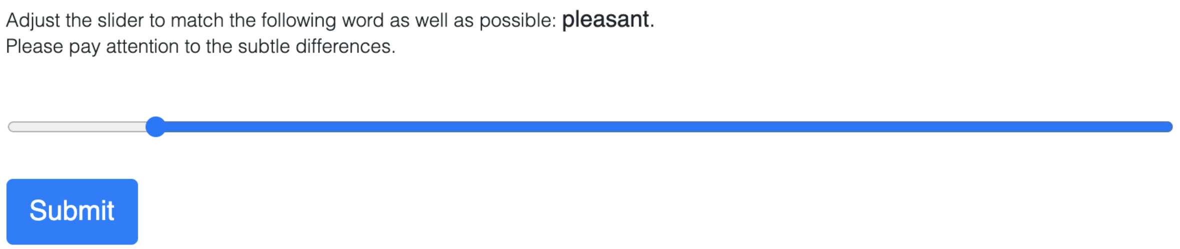

Supplementary Figure 15. Participant interface for a GSP trial.

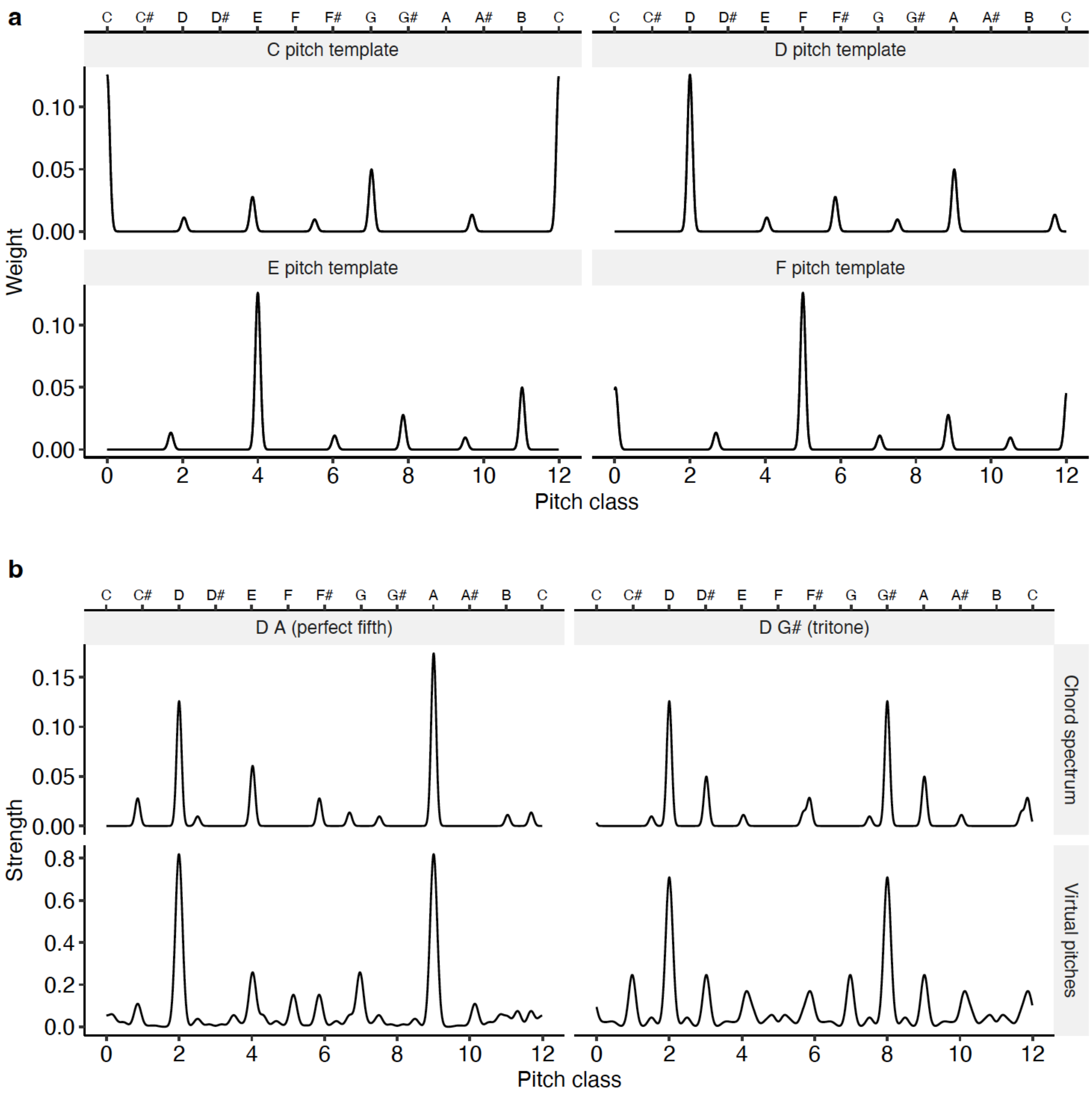

Supplementary Figure 16. The Harrison-Pearce and Milne harmonicity models. (**a**) Idealized harmonic templates corresponding to harmonic complex tones with different fundamental frequencies. (**b**) Idealized input chord spectra (top row) and corresponding virtual pitch strength (bottom row), with the latter computed as the cosine similarity between the chord spectrum and harmonic templates of different candidate pitches.

Supplementary Tables

Study	Description	Dataset	Mean ratings per participant	Total number of stimuli	Tone spectra	Participants			
						N	$N_{f/m/o}$	μ_{mus}	σ_{mus}
1A	Harmonic dyads	*dyh3dd*	37.9	7,500	Type I ($\rho = 3$)	198	73/123/2	4.1	6.9
1B(i)	Piano dyads	*harpno*	74.2	15,000	Type VI (piano) and I ($\rho = 3$)	202	78/120/4	3.9	6.3
1B(ii)	Guitar dyads	*hargtr*	71.4	15,000	Type VI (guitar) and I ($\rho = 3$)	210	86/121/3	4.5	6.9
1B(iii)	Flute dyads	*harflt*	78.9	15,000	Type VI (flute) and I ($\rho = 3$)	190	62/124/4	4.2	6.6
2A(i)	Stretched dyads	*dys3dd*	38.7	7,500	Type II ($\rho = 3, \gamma = 2.1$)	194	67/125/2	3.6	6.2
2A(ii)	Compressed dyads	*dyc3dd*	37.1	7,500	Type II ($\rho = 3, \gamma = 1.9$)	202	71/130/1	4.2	6.7
2B(i)	Harmonic dyads (Korean)	*korean-dyad-harm*	174.5	4,188	Type I ($\rho = 3$)	24	12/12/0	2.1	2.7
2B(ii)	Stretched dyads (Korean)	*korean-dyad-str*	198.1	3,961	Type II ($\rho = 3, \gamma = 2.1$)	20	10/10/0	2.2	2.9
2B(iii)	Compressed dyads (Korean)	*korean-dyad-comp*	199.1	4,777	Type II ($\rho = 3, \gamma = 1.9$)	24	12/12/0	2.3	2.8
2C	Bonang dyads	*gamdyrt*	44.1	7,500	Type I + V	170	71/97/2	5.0	7.0
3	Dyads with varying roll-off	*rodyrt*	46.6	15,000	Type I ($0 \leq \rho \leq 15$)	322	145/174/3	4.0	6.0
4A(i)	Dyads with 5 equal harmonics	*w3rdd*	78.9	11,754	Type IV_{+}	149	56/90/3	3.3	5.2
4A(ii)	Dyads without third harmonic	*wo3rdd*	75	12,000	Type IV_{-}	160	74/86/0	3.6	5.5

4A(iii)	Pure dyads	*purdyrt*	42.6	7,500	Type III	176	67/105/4	4.2	6.2
4B(i)	Major third (harmonics)	*tun3p9*	49.8	11,796	Type I ($\rho = 3$)	237	99/135/3	3.7	5.9
4B(ii)	Major third (no harmonics)	*tunp39*	49.8	13,250	Type III	266	118/144/4	3.8	6.9
4B(iii)	Major sixth (harmonics)	*tun8p9*	49.6	11,397	Type I ($\rho = 3$)	230	101/125/4	4.4	7.3
4B(iv)	Major sixth (no harmonics)	*tunp89*	49.9	11,346	Type III	227	95/131/1	3.8	6.8
4B(v)	Octave (harmonics)	*tunoch*	76.5	15,000	Type I ($\rho = 3$)	196	78/112/6	4.1	7.3
4B(vi)	Octave (no harmonics)	*tunocp*	78.2	14,471	Type III	185	68/111/6	3.1	5.7

Supplementary Table 1. Overview of dense rating experiments. This includes (left to right): Study ID, description, dataset ID, number of ratings per participant, total number of stimuli used, tone spectra of the stimuli, N the total number of participants, $N_{f/m/o}$ the number of self-reported female/male/other participants, and μ_{mus}/σ_{mus} the mean/standard deviation of reported years of musical experience.

Study	Description	Dataset	Iterations	Chains	Tone spectra	Participants			
						N	$N_{f/m/o}$	μ_{mus}	σ_{mus}
5A	Harmonic triads	*trdh3d*	40	200	Type I ($\rho = 3$)	228	86/141/1	3.8	6.2
5B(i)	Stretched triads	*trds3d*	40	200	Type II ($\rho = 3$, $\gamma = 2.1$)	229	83/145/1	4.6	7.5
5B(ii)	Compressed triads	*trdc3d*	40	200	Type II ($\rho = 3$, $\gamma = 1.9$)	233	90/141/2	4.4	7.7

Supplementary Table 2. Overview of GSP experiments. This includes (left to right): Study ID, description, dataset ID, the number of iterations per chain (excluding the random seed), the number of chains collected, the tone spectra of the stimuli used, N the total number of participants, $N_{f/m/o}$ the number of self-reported female/male/other participants, and μ_{mus}/σ_{mus} the mean/standard deviation of reported years of musical experience.

Parameter	Value		Optimization bounds	
	Initial	Optimized	Lower	Upper
Harmonicity weight (relative to interference weight)	0.750	0.837	-1.000	1.000
Amplitude exponent in interference model	1.000	1.359	0.000	5.000
Slow-beat boundary (critical bandwidths)	0.100	0.096	0.000	1.000
Slow-beat pleasantness	1.500	1.632	0.000	5.000

Supplementary Table 3. Model parameter optimization.
