# Supplementary material for: Timbral effects on consonance disentangle psychoacoustic mechanisms and suggest perceptual origins for musical scales
Source: Nat Commun. 2024 Feb 19;15:1482. doi: 10.1038/s41467-024-45812-z (PMC11258268; doi:10.1038/s41467-024-45812-z)
Supplement: Supplementary file 3 — Description of Additional Supplementary Files [file 41467_2024_45812_MOESM3_ESM.pdf]

Description of Additional Supplementary Files

File Name: Supplementary Movie 1
Description: Dyadic pleasantness judgments for harmonic complex tones (N = 198 US participants) along with model predictions. Behavioral results are summarized using a kernel smoother with a bandwidth of 0.2 semitones, with 95% confidence intervals (bootstrapped, 1,000 replicates) shaded in gray, peak locations plotted as red circles with red rectangles indicating mean values +/- 95% confidence intervals. Dotted lines indicate the location of the compressed, harmonic, and stretched octaves.

File Name: Supplementary Movie 2
Description: Dyadic pleasantness judgments for stretched complex tones (N = 194 US participants) along with model predictions. Behavioral results are summarized using a kernel smoother with a bandwidth of 0.2 semitones, with 95% confidence intervals (bootstrapped, 1,000 replicates) shaded in gray, peak locations plotted as red circles with red rectangles indicating mean values +/- 95% confidence intervals. Dotted lines indicate the location of the compressed, harmonic, and stretched octaves.

File Name: Supplementary Movie 3
Description: Dyadic pleasantness judgments for compressed complex tones (N = 202 US participants) along with model predictions. Behavioral results are summarized using a kernel smoother with a bandwidth of 0.2 semitones, with 95% confidence intervals (bootstrapped, 1,000 replicates) shaded in gray, peak locations plotted as red circles with red rectangles indicating mean values +/- 95% confidence intervals. Dotted lines indicate the location of the compressed, harmonic, and stretched octaves.

File Name: Supplementary Movie 4
Description: Dyadic pleasantness judgments for harmonic complex tones (N = 24 South Korean participants) along with model predictions. Behavioral results are summarized using a kernel smoother with a bandwidth of 0.2 semitones, with 95% confidence intervals (bootstrapped, 1,000 replicates) shaded in gray, peak locations plotted as red circles with red rectangles indicating mean values +/- 95% confidence intervals. Dotted lines indicate the location of the compressed, harmonic, and stretched octaves.

File Name: Supplementary Movie 5
Description: Dyadic pleasantness judgments for stretched complex tones (N = 20 South Korean participants) along with model predictions. Behavioral results are summarized using a kernel smoother with a bandwidth of 0.2 semitones, with 95% confidence intervals (bootstrapped, 1,000 replicates) shaded in gray, peak locations plotted as red circles with red rectangles indicating mean

values +/- 95% confidence intervals. Dotted lines indicate the location of the compressed, harmonic, and stretched octaves.

File Name: Supplementary Movie 6
Description: Dyadic pleasantness judgments for compressed complex tones (N = 24 South Korean participants) along with model predictions. Behavioral results are summarized using a kernel smoother with a bandwidth of 0.2 semitones, with 95% confidence intervals (bootstrapped, 1,000 replicates) shaded in gray, peak locations plotted as red circles with red rectangles indicating mean values +/- 95% confidence intervals. Dotted lines indicate the location of the compressed, harmonic, and stretched octaves.

File Name: Supplementary Movie 7
Description: Pleasantness judgments for dyads comprising a harmonic complex tone (lower) combined with an idealized bonang tone (upper) (N = 170 US participants) along with model predictions. Behavioral results are summarized using a kernel smoother with a bandwidth of 0.2 semitones, with 95% confidence intervals (bootstrapped, 1,000 replicates) shaded in gray, peak locations plotted as red circles with red rectangles indicating mean values +/- 95% confidence intervals. The slendro scale, approximated as 5-tone equal temperament, is plotted with dashed lines.

File Name: Supplementary Movie 8
Description: Dyadic pleasantness judgments as a function of roll-off (N = 322 US participants) for harmonic dyads with 12, 7, and 2 dB/octave roll-off values along with model predictions. Behavioral results are summarized using a kernel smoother with a bandwidth of 0.2 semitones, with 95% confidence intervals (bootstrapped, 1,000 replicates) shaded in gray, peak locations plotted as red circles. Audio corresponds to the 2dB/octave condition.

File Name: Supplementary Movie 9
Description: Dyadic pleasantness judgments as a function of roll-off (N = 322 US participants) for harmonic dyads with 12, 7, and 2 dB/octave roll-off values along with model predictions. Behavioral results are summarized using a kernel smoother with a bandwidth of 0.2 semitones, with 95% confidence intervals (bootstrapped, 1,000 replicates) shaded in gray, peak locations plotted as red circles. Audio corresponds to the 7dB/octave condition.

File Name: Supplementary Movie 10
Description: Dyadic pleasantness judgments as a function of roll-off (N = 322 US participants) for harmonic dyads with 12, 7, and 2 dB/octave roll-off values along with model predictions. Behavioral results are summarized using a kernel smoother with a bandwidth of 0.2 semitones, with 95% confidence intervals (bootstrapped, 1,000 replicates) shaded in gray, peak locations plotted as red circles. Audio corresponds to the 12dB/octave condition.

File Name: Supplementary Movie 11
Description: Dyadic pleasantness judgments for complex tones with 5 equal harmonics (N = 149 US participants) along with model predictions. Behavioral results are summarized using a kernel smoother with a bandwidth of 0.2 semitones, with 95% confidence intervals (bootstrapped, 1,000 replicates) shaded in gray, peak locations plotted as red circles with red rectangles indicating mean values +/- 95% confidence intervals.

File Name: Supplementary Movie 12
Description: Dyadic pleasantness judgments for complex tones with 5 equal harmonics and the 3rd harmonic deleted (N = 160 US participants) along with model predictions. Behavioral results are summarized using a kernel smoother with a bandwidth of 0.2 semitones, with 95% confidence intervals (bootstrapped, 1,000 replicates) shaded in gray, peak locations plotted as red circles with red rectangles indicating mean values +/- 95% confidence intervals.

File Name: Supplementary Movie 13
Description: Dyadic pleasantness judgments for pure tones (N = 176 US participants) along with model predictions. Behavioral results are summarized using a kernel smoother with a bandwidth of 0.2 semitones, with 95% confidence intervals (bootstrapped, 1,000 replicates) shaded in gray, peak locations plotted as red circles with red rectangles indicating mean values +/- 95% confidence intervals.

File Name: Supplementary Movie 14
Description: Dyadic pleasantness judgments for (mis)tunings of the major 3rd produced using harmonic complex tones (N = 237 US participants). Behavioral results are summarized using a kernel smoother with a bandwidth of 0.035 semitones, with 95% confidence intervals (bootstrapped, 1,000 replicates) shaded in gray. Just-intoned and equal-tempered versions of the interval are marked with solid and dashed vertical lines, respectively.

File Name: Supplementary Movie 15
Description: Dyadic pleasantness judgments for (mis)tunings of the major 6th produced using harmonic complex tones (N = 230 US participants). Behavioral results are summarized using a kernel smoother with a bandwidth of 0.035 semitones, with 95% confidence intervals (bootstrapped, 1,000 replicates) shaded in gray. Just-intoned and equal-tempered versions of the interval are marked with solid and dashed vertical lines, respectively.

File Name: Supplementary Movie 16
Description: Dyadic pleasantness judgments for (mis)tunings of the octave produced using harmonic complex tones (N = 196 US participants). Behavioral results are summarized using a kernel smoother with a bandwidth of 0.035 semitones, with 95% confidence intervals (bootstrapped, 1,000 replicates) shaded in gray. Just-intoned and equal-tempered versions of the interval are marked with solid and dashed vertical lines, respectively.

File Name: Supplementary Movie 17
Description: Dyadic pleasantness judgments for (mis)tunings of the major 3rd produced using pure tones (N = 266 US participants). Behavioral results are summarized using a kernel smoother with a bandwidth of 0.035 semitones, with 95% confidence intervals (bootstrapped, 1,000 replicates) shaded in gray. Just-intoned and equal-tempered versions of the interval are marked with solid and dashed vertical lines, respectively.

File Name: Supplementary Movie 18
Description: Dyadic pleasantness judgments for (mis)tunings of the major 6th produced using pure tones (N = 227 US participants). Behavioral results are summarized using a kernel smoother with a bandwidth of 0.035 semitones, with 95% confidence intervals (bootstrapped, 1,000 replicates) shaded in gray. Just-intoned and equal-tempered versions of the interval are marked with solid and dashed vertical lines, respectively.

File Name: Supplementary Movie 19
Description: Dyadic pleasantness judgments for (mis)tunings of the octave produced using pure tones (N = 185 US participants). Behavioral results are summarized using a kernel smoother with a bandwidth of 0.035 semitones, with 95% confidence intervals (bootstrapped, 1,000 replicates) shaded in gray. Just-intoned and equal-tempered versions of the interval are marked with solid and dashed vertical lines, respectively.
