# Supplementary material for: Timbral effects on consonance disentangle psychoacoustic mechanisms and suggest perceptual origins for musical scales
Source: Nat Commun. 2024 Feb 19;15:1482. doi: 10.1038/s41467-024-45812-z (PMC11258268; doi:10.1038/s41467-024-45812-z)
Supplement: Supplementary file 5 — Reporting Summary [file 41467_2024_45812_MOESM5_ESM.pdf]

nature portfolio

Corresponding author(s): Raja Marjieh, Peter M. C. Harrison, Nori Jacoby

Last updated by author(s): Nov 24, 2023

Reporting Summary

Nature Portfolio wishes to improve the reproducibility of the work that we publish. This form provides structure for consistency and transparency in reporting. For further information on Nature Portfolio policies, see our Editorial Policies and the Editorial Policy Checklist.

Statistics

For all statistical analyses, confirm that the following items are present in the figure legend, table legend, main text, or Methods section.

n/a	Confirmed	
☐	☒	The exact sample size (*n*) for each experimental group/condition, given as a discrete number and unit of measurement
☐	☒	A statement on whether measurements were taken from distinct samples or whether the same sample was measured repeatedly
☐	☒	The statistical test(s) used AND whether they are one- or two-sided<br>*Only common tests should be described solely by name; describe more complex techniques in the Methods section.*
☒	☐	A description of all covariates tested
☐	☒	A description of any assumptions or corrections, such as tests of normality and adjustment for multiple comparisons
☐	☒	A full description of the statistical parameters including central tendency (e.g. means) or other basic estimates (e.g. regression coefficient) AND variation (e.g. standard deviation) or associated estimates of uncertainty (e.g. confidence intervals)
☐	☒	For null hypothesis testing, the test statistic (e.g. F, t, r) with confidence intervals, effect sizes, degrees of freedom and P value noted<br>*Give P values as exact values whenever suitable.*
☒	☐	For Bayesian analysis, information on the choice of priors and Markov chain Monte Carlo settings
☒	☐	For hierarchical and complex designs, identification of the appropriate level for tests and full reporting of outcomes
☐	☒	Estimates of effect sizes (e.g. Cohen's *d*, Pearson's *r*), indicating how they were calculated

Our web collection on statistics for biologists contains articles on many of the points above.

Software and code

Policy information about availability of computer code

Data collection	All data was collected using PsyNet (v3.0.0; https://www.psynet.dev), our in-house framework for complex experiment design. This framework builds on Dallinger (v7.6.0; https://www.dallinger.readthedocs.io), a platform for experiment hosting and deployment. We released all code for reproducing the experiments in a dedicated repository (https://gitlab.com/raja.marjieh/consonance-and-timbre-data) as well as the following public OSF repository (https://osf.io/83w2b/; DOI 10.17605/OSF.IO/83W2B). To synthesize tones online in the participant browser we used the Tone.js JavaScript library (v14.7.77; https://tonejs.github.io/).
Data analysis	All data analysis was conducted using R (v4.2.1) and the code is made publicly available in a dedicated repository (https://gitlab.com/pmcharrison/timbre-and-consonance-paper). We used R together with a collection of 181 packages which are listed in full (including version numbers) in the renv.lock file in the code repository. In addition, we supplemented our analysis code with an interactive app that facilitates model exploration (https://pmcharrison.gitlab.io/timbre-and-consonance-paper/supplementary.html).

For manuscripts utilizing custom algorithms or software that are central to the research but not yet described in published literature, software must be made available to editors and reviewers. We strongly encourage code deposition in a community repository (e.g. GitHub). See the Nature Portfolio guidelines for submitting code & software for further information.

Data

Policy information about availability of data

All manuscripts must include a data availability statement. This statement should provide the following information, where applicable:

- Accession codes, unique identifiers, or web links for publicly available datasets
- A description of any restrictions on data availability
- For clinical datasets or third party data, please ensure that the statement adheres to our policy

All data generated in this work have been deposited in a publicly available OSF repository under the following URL:
https://osf.io/83w2b/ (DOI 10.17605/OSF.IO/83W2B)

To further facilitate the process, data can also be explored and exported via the following interactive web app:
https://pmcharrison.gitlab.io/timbre-and-consonance-paper/supplementary.html

Naturalistic instrument tone samples used in this study are available at the Midi.js Soundfont database
https://github.com/gleitz/midi-js-soundfonts

Benchmark behavioral datasets for Study 1 (Schwartz et al., 2003; Bowling et al., 2018; McPherson et al., 2020) are available through the following repository:
https://gitlab.com/pmcharrison/timbre-and-consonance-paper

Research involving human participants, their data, or biological material

Policy information about studies with human participants or human data. See also policy information about sex, gender (identity/presentation), and sexual orientation and race, ethnicity and racism.

Reporting on sex and gender

Gender and sex were not part of our study design as we were interested in the overall population level effects of timbre on consonance perception. Overall, of the US participants recruited on MTurk (N=4,204) 39.7% self-identified as female, 59.0% as male and 1.3% as other. As for the South Korean participants (N=68), half of them self-identified as female and the other half as male. A more detailed breakdown for each individual experiment can be found in the Supplementary Information (Tables S1-2) and the data repository provided above.

Reporting on race, ethnicity, or other socially relevant groupings

We did not group our participants based on race / ethnicity or other socially relevant groupings. The only distinction was based on self-reported global geographic location, namely, United States and South Korea (see population characteristics below).

Population characteristics

We collected data from two online participant cohorts based on geographic location, namely, United States (N=4,204) and South Korea (N=68). In the US cohort, reported ages varied in the range 18-81 (M=37.5, SD=11.0) and self-reported years of musical experience were in the range 0-55 (M=4, SD=6.6). As for the South Korean cohort, the reported age statistics were (M=27, SD=10.50) and those of the years of musical experience were (M=2.19, SD=2.67). Additional details for each individual experiment are provided in the Methods Section.

Recruitment

The US cohort was recruited from Amazon Mechanical Turk (AMT), a well-established online crowd-sourcing platform. We specified the following recruitment criteria: that participants must be at least 18 years of age, that they reside in the United States, and that they have a 95% or higher approval rate on previous AMT tasks. The South Korean cohort was recruited through a research assistant residing in the local area (Lee et al., 2021); AMT was not possible in this case as AMT does not currently run in South Korea. Participants were required both to be born in South Korea and to be current residents there.

Ethics oversight

All participants provided informed consent in accordance with the Max Planck Society Ethics Council approved protocols 2020_05 (US) and 2021_42 (South Korea).

Note that full information on the approval of the study protocol must also be provided in the manuscript.

Field-specific reporting

Please select the one below that is the best fit for your research. If you are not sure, read the appropriate sections before making your selection.

☐ Life sciences ☒ Behavioural & social sciences ☐ Ecological, evolutionary & environmental sciences

For a reference copy of the document with all sections, see nature.com/documents/nr-reporting-summary-flat.pdf

Behavioural & social sciences study design

All studies must disclose on these points even when the disclosure is negative.

Study description

We conducted 5 quantitative studies comprising 23 large-scale online experiments aimed at studying the effect of timbre on musical consonance perception. These studies fall into two experimental paradigms, a) Dense rating, whereby participants listen and rate the pleasantness of various two-tone chords sampled uniformly and continuously from a specified intervallic range, and b) Gibbs

Sampling with People (Harrison et al. 2020), whereby participants iteratively manipulate the intervals of three-note chords using sliders to optimize for their acoustic pleasantness (see Methods for full details).

Research sample	Our participants comprised two populations. The larger group (N=4,204) were United States participants recruited on Amazon Mechanical Turk. We chose US participants as our main population to ensure online data quality and efficient large scale recruitment. As an additional cross-cultural control, we collected data from a second sample of South Korean participants (N=68). See Population Characteristics and Recruitment Sections above for demographic details and recruitment criteria.
Sampling strategy	See Recruitment Section above for recruitment criteria. We ran each online experiment for about a day, targeting about 150-200 participants for 1D experiments, and targeting somewhat larger cohorts (~200-350) for the multi-dimensional and tuning experiments (see Methods); the latter experiments required exploring larger stimulus spaces and/or more subtle perceptual effects. These target numbers were established via pilot experiments and verified post-hoc using Monte Carlo split-half reliability analyses, which indicated an excellent reliability for both the dyad paradigm (r = .87, 95% CI: [.74, .94]) and the triad paradigm (r = .93, 95% CI: [.91, .96]).
Data collection	All experiments were implemented using PsyNet (https://www.psynet.dev), our in-house framework for complex experiment design (Harrison et al., 2020). This framework builds on Dallinger, a platform for experiment hosting and deployment. Participants engage anonymously with the experiment through a front-end interface displayed in the web browser, which communicates with a back-end Python server cluster that organizes the experiment timeline. The cluster is managed by the web service Heroku which orchestrates a collection of virtual instances that share the experiment management workload, as well as an encrypted Postgres database instance for data storage.
Timing	Data was collected over the course of 8 months between November 2020 and June 2021.
Data exclusions	To ensure data quality, we excluded participants who either failed to pass the initial headphone check (see Methods for details) or failed to complete the experiment due to technical errors / early drop out. These participants were automatically labeled as "failed" by our experimental framework and were excluded from the studies. Overall, we excluded 2,373 participants.
Non-participation	Of the 6,645 online participants that attempted our experiments on AMT, 2,373 dropped out due to technical errors, failure to pass the initial headphone check or other personal reasons (in all cases these were automatically labeled as "failed" by our system). No participants declined participation.
Randomization	N/A, participants were not allocated into experimental groups beyond the overall division of United States vs. South Korean cohorts.

Reporting for specific materials, systems and methods

We require information from authors about some types of materials, experimental systems and methods used in many studies. Here, indicate whether each material, system or method listed is relevant to your study. If you are not sure if a list item applies to your research, read the appropriate section before selecting a response.

Materials & experimental systems

n/a	Involved in the study
☒	☐ Antibodies
☒	☐ Eukaryotic cell lines
☒	☐ Palaeontology and archaeology
☒	☐ Animals and other organisms
☒	☐ Clinical data
☒	☐ Dual use research of concern
☒	☐ Plants

Methods

n/a	Involved in the study
☒	☐ ChIP-seq
☒	☐ Flow cytometry
☒	☐ MRI-based neuroimaging
